# Site-Independent Mapping of Clay Content in Vineyard Soils via Mobile Proximal Gamma-Ray Spectrometry and Machine Learning Calibrations

**DOI:** 10.3390/s24144528

**Published:** 2024-07-12

**Authors:** Ralf Wehrle, Stefan Pätzold

**Affiliations:** Institute of Crop Science and Resource Conservation (INRES)—Soil Science and Soil Ecology, University of Bonn, D-53115 Bonn, Germany

**Keywords:** proximal soil sensing, gamma spectroscopy, soil heterogeneity, precision agriculture, viticulture, soil texture, Random Forest

## Abstract

Vineyards hold considerable soil variability between regions and plots, and there is frequently large soil heterogeneity within plots. Clay content in vineyard soils is of interest with respect to soil management, environmental monitoring, and wine quality. However, spatially resolved clay mapping is laborious and expensive. Gamma-ray spectrometry (GS) is a suitable tool for predicting clay content in precision agriculture when locally calibrated, but it has scarcely been tested site-independently and in vineyards. This study evaluated GS to predict clay content with a site-independent calibration and four machine learning algorithms (Support Vector Machines, Random Forest, k-Nearest Neighbors, and Bayesian regulated neuronal networks) in eight vineyards from four German vine-growing regions. Clay content in the studied soils ranged from 62 to 647 g kg^−1^. The Random Forest calibration was most suitable. Test set evaluation revealed good model performance for the entire dataset with RPIQ = 4.64, RMSE_P_ = 56.7 g kg^−1^, and R^2^ = 0.87; however, prediction quality varied between the sites. Overall, GS with the Random Forest model calibration was appropriate to predict the clay content and its spatial distribution, even for heterogeneous geopedological settings and in individual plots. Therefore, GS is considered a valuable tool for soil mapping in vineyards, where clay content and product quality are closely linked.

## 1. Introduction

Clay content is a key soil property that has an impact on a variety of soil functions such as soil water balance, nutrient stock, nutrient availability, aggregation, and stabilization, and respective storage of organic carbon [1]. Storage of organic carbon has recently been addressed for the specific conditions in precision viticulture [2]. However, clay content can be spatially variable, even at the plot scale. In the context of precision agriculture, high-resolution information on soil properties is needed to establish site-specific and sustainable management practices [3]. Therefore, clay content forms an important parameter to be determined accurately and spatially resolved. This holds particularly true for vine, a high-value perennial crop, where soil properties significantly impact the quality of the harvest [4,5]. Yet, at present, conventional methods of mapping soil properties are laborious, costly, and often fail to provide the required spatial resolution [6]. Here, mobile gamma-ray spectrometry (GS) is becoming a recognizable technology for proximal soil sensing applications [7]. For GS, the natural gamma quanta emitting radionuclides of arable soils can be correlated to the soil properties of the approximately uppermost 30 to 40 cm of soil [8,9]. The different features of the gamma spectrum are influenced by geological, mineralogical, and pedological factors (i.e., the geopedological setting) and can therefore be related to the sand, silt, and clay contents of soils [10,11,12]. During rock weathering and pedogenesis, radionuclides can remain within the soil, adsorbed by clay minerals, iron oxides, and organic matter. In contrast, factors such as soil pH, moisture, and internal drainage can increase the leaching process, which in turn leads to radionuclide loss from topsoil [9]. Moreover, soil erosion leads to the loss or the re-distribution of topsoil material from slopes to deeper relief positions. These pedogenic processes result in the varying distribution of radionuclides in the soil and within fields or vineyards. For this reason, proximal GS has been successfully used for mapping soil texture at single sites [7].

Use of mobile GS in soil science relies on the detection of the naturally occurring elements Potassium-40 (K-40), Uranium-238 (U-238), and Thorium-232 (Th-232). While K-40 is measured directly, the gamma-ray-emitting decay products Bismuth-214 and Thallium-208, from the respective decay series, are monitored to quantify U-238 and Th-232, respectively. Gamma-ray spectrometers record the counts per second (cps) in the following energy ranges (regions of interest (ROI) or “windows”): 1.37–1.57 MeV for K-40, 1.66–1.86 MeV (Bi-214) for U-238 measurement, and 2.41–2.81 MeV (Tl-208) for Th-232 measurement. Furthermore, the total gamma counts (TC) in the energy window 0.4–2.81 MeV provide useful information [9]. Besides Bi-214, Radon-222 (Rn-222) occurs in the U-238 decay series and seriously impacts the measurements. Since Rn-222 is a gas, it can emanate from the soil and be translocated in the atmosphere, thereby changing its spatial distribution. Precipitation can return it to the soil far from its source, so that its spatial relationship to soil properties is distorted when recording localized gamma spectra [9].

Soil moisture attenuates the gamma radiation measured at the soil surface; however, this influence can be corrected by a linear factor (10% moisture content attenuates the signal by 10%) [9]. This factor is used widely to correct the gamma signal at variable soil moisture conditions [7,10,11,12,13]. However, the mentioned attenuation factor for soil moisture does not generally apply for Rn-222, because Rn-222 counts can even increase with increasing soil moisture [9].

Mobile GS can be conducted while driving (on-the-go) or during stationary measurements (stop-and-go). Both types yielded satisfactory results for the calibration on soil texture [12]. Remote and proximal GS are capable of detecting spatial soil texture patterns from the field scale to the landscape scale [13,14]. However, calibrations are only valid for singe fields [15] or a limited number of sites with similar geopedological settings [16]. For sample sets containing various sites and heterogeneous geopedological settings, the conditions become more complex and related studies are relatively scarce. This is because the relationship between gamma spectrum and soil texture is only of an indirect nature. The correlation between the different grain size fractions and gamma nuclide content in soil (e.g., clay and K-40) is determined by various factors as pointed out above, notably soil mineralogical composition. For example, ref. [12] showed a close relation between soil clay content and TC in two fields in Western Germany, but they had an opposite orientation due to contrasting geopedological settings. As a consequence, linear calibrations often fail when multiple fields with varying geopedological backgrounds are included in the model.

Machine learning (ML) methods may outperform linear regression approaches for calibrating models on soil data; this is because ML algorithms are capable of determining complex and non-linear relationships [17]. Various ML algorithms exist; however, only a few have been applied to GS calibrations so far. The performances of artificial neuronal networks and Support Vector Machines (SVMs) were compared for single field applications with promising results for both calibration approaches [10]. The potential of non-linear SVM for the calibration of site-independent prediction models for soil texture over varying geopedological settings was shown by [11]. Yet, Ref. [12] proved that the models lack the robustness required for sites that are not included in the calibration, even despite similar geopedological settings, thus showing problems in transferability. The different ML methods rely on varying mathematical approaches to make estimations and the efficiency of different ML algorithms is highly dependent on the dataset and the provided explanatory information [18]. For building texture models on GS data, different ML approaches are potentially feasible, but up to now, there has been no general accordance on which algorithm performs best for GS. Therefore, a comparison of different ML approaches is still required to find the optimal algorithm for a specific dataset, particularly when it comes to site-independent calibrations. In recent years, there has been a decrease in the use of methods such as SVM, and more established methods such as Random Forest (RF) have emerged. This approach has seen accelerated popularity in soil science including mapping and spectroscopy [18]. Yet, the superiority of RF compared to other established ML methods for site-independent GS soil texture models is not proven. In this study, four current ML algorithms were applied to build a site-independent prediction model for clay content in soil. The tested ML approaches are the following: Support Vector Machines (SVMs), Random Forest (RF), k-Nearest Neighbors (KNNs), and Bayesian regulated neuronal networks (BNNs). An overview of the different algorithms is provided in Section 2.5.

To create soil maps from a limited number of sampling points, spatial interpolation is a common approach. Deterministic methods such as Inverse Distance Weighting or Splines tend to lead to oversimplification because spatial autocorrelation is not considered [19]. Therefore, researchers rely mostly on methods such as Kriging, where spatial autocorrelation is a prerequisite for interpolation [20]. For this purpose, experimental variograms have to be created and interpreted. Depending on the variogram results, soil maps created by spatial interpolation always come with a varying amount of uncertainty. As an alternative to interpolation, area-wide sensor data can be directly used for precision agriculture without further processing, if data density matches the final application needs [12]. Mobile GS on-the-go measurements can be carried out in real time and provide spatial, highly resolved data [21]. Depending on measurement geometry (detector crystal type and size, distance to surface, etc.), the footprint of proximal GS varies; for a common approach, like that published by [11,12], it approximates a 2 m radius around the detector crystal [22]. Therefore, on-the-go GS bears the advantage that the observed values do not need to be interpolated, resulting in fewer methodological assumptions which can simplify application in precision agriculture. Therefore, this study further focused on using the calibrated site-independent GS model for soil mapping purposes by creating clay prediction maps of on-the-go gamma measurements without the necessity of spatial interpolation methods.

In summary, the aims of this study were as follows: (I) to show that proximal GS can predict soil clay content; (II) to test the performance of RF calibrations to other state-of-the-art ML calibrations for a dataset of eight vineyards with varying geopedological settings; (III) to evaluate the RF approach on the field-scale; (IV) to create soil clay maps by RF calibrations and GS on-the-go measurements.

## 2. Materials and Methods

### 2.1. Characterization of the Study Sites

Eight vineyards in different wine-growing areas in Rhineland-Palatinate (Germany) were investigated in this study. As shown in Table 1, the geopedological setting differed remarkably between the studied fields.

Soils in Siebeldingen were either developed from loess (Sieb B) or from Pleistocene periglacial slope deposits (ppsd) from weathered Keuper and Bunter Sandstone sedimentary rocks, partly covered with ex situ loess as part of a slope adjustment (Sieb N). In Sprendlingen (Rhine-Hesse), soils in both examined fields (Spre B, Spre N) originated from weathered Oligocene marl. The vineyard Kanzem consisted of weathering products of Devonian sandstones and shales. The Leiwen sites were located in the Moselle Region (Leiw H, Leiw K) and the site Ruppertsberg near Neustadt/W. (Rupp) in the Palatinate vine growing area. These soils developed from Pleistocene fluvial deposits originating from different river catchments.

### 2.2. Determination of Ground Truth Data

To explore the full extent of clay content variability, the study sites were systematically sampled for reference analyses (“ground truth”). Preliminary gamma spectrometry surveys were conducted for pattern recognition and subsequent reference sampling, with the same spectrometer setup as described in Section 2.3, but “on-the-go” at 0.7–1.4 m s^−1^ driving velocity. All soil samples were taken from the topsoil with an Edelman drill with three drills per sampling point. Clay content was determined as part of the texture analysis by the combined sieve and pipette method [23]. Soil moisture content was determined gravimetrically in order to correct the gamma counts with the IAEA factor [9].

### 2.3. Stop-and-Go Gamma Measurements

**Figure 1:**
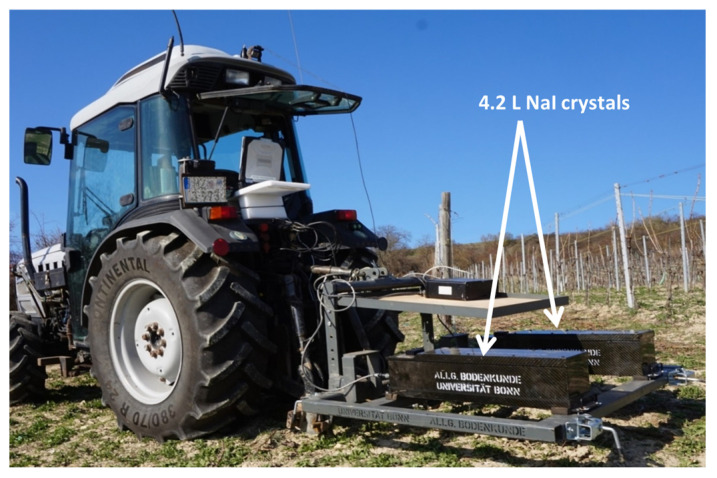
Tractor-mounted gamma spectrometer.

The gamma-ray spectrometer used in this study (type “RSX-1”) was manufactured by Radiation Solutions Inc. (Mississauga, ON, Canada). It consisted of two 4.2 l NaI(Tl) crystals which were mounted to the tractor’s three-point linkage via a steel frame that has been constructed at the University of Bonn for this purpose. The two crystals were linked with an advanced digital spectrometer (ADS) console so that the captured gamma decay energy pulses were synchronously processed and transformed into a spectrum with 1024 channels (Figure 1). Positioning data were provided by an internal GPS module with an external antenna.

All measurements were carried out at 1 Hz frequency and at 0.3 m distance to the soil surface. The spectra were recorded in two modes: (i) in a stationary mode (“stop-and-go”) and (ii) while driving (“on-the-go”). These had identical geometric measurement setups. To rule out the immanent signal noise, the stop-and-go spectra were taken at the reference sampling points and averaged over one minute recording time, resulting in 60 spectra per sampling point, as described in [12]. Descriptive statistics of conventionally measured texture fractions, cps for the ROIs, and field metadata are given in Table 2. In this study, U-238 was not evaluated with respect to the above-mentioned problems related to Rn-222. The largely varying clay content of the test sites in combination with the different measurement dates (i.e., soil moisture conditions) led to this decision. Depending on the field size and the extent of heterogeneity, 10–53 points per study site were sampled. The overall clay contents ranged from approximately 6 to 65% clay, thus covering the prevailing range in German vineyard soils. The coefficient of variation (coeff. var.) for clay as indicator of extent of heterogeneity also differed remarkably between the study sites.

### 2.4. Spatial Predictions of on-the-go Gamma Measurements

Recording gamma spectra while driving (on-the-go) combines several sources of error that have to be encountered when evaluating the data. First, gamma decay is not deterministic, but reveals variability over short periods of time. Therefore, two subsequent spectra will always differ, even on homogeneous ground. Second, recording spectra at 1 Hz rate while driving at 0.7 m s^−1^ results in 1.4 spectra per meter travelled distance. Third, the signal origin (i.e., the area from which the gamma counts are collected, “footprint”) has no sharp boundary. With increasing distance from the detector crystals, the relative signal contribution decreases due to the non-directional emission of quanta from soil. For the chosen geometric setup (see Section 2.3), the footprint amounts to approximately 2 m radius around the detector crystals [12,22]. Therefore, neighbored spectra recorded on-the-go reveal a significant footprint overlap that cannot be precisely quantified. Finally, soil heterogeneity over very small distances (i.e., at meter scale) results, if it is present, in a small-scale variability of gamma quanta emission. In this study, a moving-window approach, as proposed by [12], was applied in order to take the various sources of uncertainty into account and to smooth the data. A spectra alignment along the driving lanes was predetermined with respect to the linear orientation of cane-trained vineyards. Five subsequent spectra were taken to calculate mean values for each ROI. Note that each spectrum was considered in five mean values; this means that the number of data points was not reduced by smoothing. After calibrating ML models for clay upon stop-and-go spectra (following Section 2.5 and Section 2.6), the smoothed on-the-go gamma spectra were used to predict the clay content for the whole fields.

### 2.5. Tested Machine Learning Methods

Among the large number of supervised ML algorithms that are available [17], four state-of-the-art ML approaches were selected for this study.

K-Nearest Neighbors (KNN) is a simple and non-parametric machine learning algorithm. The main concept of KNN is that near observations in the feature space are more closely related than distant ones. Thus, within the feature space, predictions are performed based on neighborhood, which is defined by the k-number of training observations, located nearest to the predicted point [24]. In short, the KNN regression algorithm works as follows: First, the Euclidean (or Mahalanobis) distance from the target sample to the known samples is computed. Second, the known samples are ordered by increasing distance. Then, the optimal number k of Nearest Neighbors, based on root mean squared error (RMSE) and cross-validation (CV), is found. Finally, an inverse distance-weighted average with the k-nearest multivariate neighbors is calculated.

Neuronal networks (NNs) are established ML methods for regression and classification problems in a broad spectrum of science disciplines. The structure of an NN consists of interconnected units (neurons) which estimate the non-linear correlation between each variable. The predictor variables (input neurons) are connected to a single layer or multiple layers of hidden neurons, which are then linked to the target variable [25]. As NN is part of the deep learning segment of ML, it is known to be susceptible to overfitting for smaller datasets [18]. Therefore, the Bayesian regulated NN (BNN), that fits a two-layered neuronal network [26,27], was used in this study. Bayesian training of NN obtains a range of weights, which in turn gives a distribution of predicted values rather than a single value, thus explicitly accounting for the uncertainty in the predictions. Bayesian training therefore prevents the overfitting of the NN. As such, BNNs incorporate the advantages of conventional NNs while eliminating some of the drawbacks.

The Random Forest (RF) approach is a learning algorithm that uses multiple decision trees for training and the results are based on the predictions from an ensemble of the individual trees. The RF regression process can be divided into three parts. First, several sub-sets (bootstrap samples) are randomly drawn from the original dataset with replacement. The second part is the construction of sub-decision trees on each sub-dataset and the output of respective regression results. Finally, the predictions of individual trees are averaged to obtain the final prediction. As a result of combining individual decision trees, RF is able to maintain a high model robustness, even when calibrated with smaller datasets with few explanatory variables [28].

Support Vector Machines (SVMs) comprise a supervised, non-parametric, non-linear statistical learning method [29]. When classes of objects are not separable with a linear classifier, the ML method has proven to be an appropriate tool for predictive regression modeling. The objects’ coordinates are rearranged in a higher dimensional feature space; thereby, the number of dimensions can be infinite [30]. So-called kernels are used to compute the classification hyperplane in a high-dimensional feature space. Kernels are mathematical functions that operate in the input space and move the data in the feature space. For this study, the radial basis function kernel was used.

### 2.6. Machine Learning Model Calibration

Before model calibration, the dataset was split into 80% training and 20% testing samples. As predictor variables, the gamma ROIs for TC, K-40, and Th-232, as well as the Th-232/K-40-ratio, were used. All algorithms were calibrated using repeated 10-fold CV. Cross-validation was optimized by an automated grid search for the specific hyperparameters of each algorithm. For SVM, the hyperparameters “sigma” and “cost” were used. The KNN calibrations were conducted by the parameter “neighbors”, BNN by “Neurons”, and RF by “mtry”. Then, a test set validation (TSV; 20% testing samples) was performed to test the model performance on independent samples. The different ML calibrations were performed with the statistic software R (2013) using the Classification and Regression Training (Caret) package [31].

The quality of the predictive models was characterized by different parameters in accordance to [32]: the coefficient of determination of cross-validation (R^2^_CV_), the coefficient of determination of prediction (R^2^_Pr_; for TSV), the root mean squared error of cross validation (RMSE_CV_), the root mean squared error of prediction (RMSE_Pr_), the ratio of performance to interquartile distance of cross-validation (RPIQcv), and the ratio of performance to interquartile distance of prediction (RPIQ_Pr_). These parameters were calculated it as follows:

(1) $$R^{2}=\frac{\sum_{i=1}^{n}{\left(f_{i}-\bar{y}\right)}^{2}}{\sum_{i=1}^{n}{\left(y_{i}-\bar{y}\right)}^{2}}$$

(2) $$\mathrm{RMSE}=\sqrt{\frac{1}{n}\overset{n}{\underset{i=1}{\sum }}{\left(f_{i}-y_{i}\right)}^{2}}$$

(3) $$\mathrm{RPIQ}=\frac{\mathrm{IQ}}{\mathrm{RMSE}}$$

where f_i_ is the predicted value, y_i_ is the respective observed value, and IQ is the interquartile distance that gives the range that accounts for 50% of the population around the median. For RPIQ values, the threshold for an unsuccessful model performance was defined by RPIQ < 2.5, according to [33].

## 3. Results and Discussion

### 3.1. Variability of Gamma Features and Geopedological Setting

**Figure 2:**
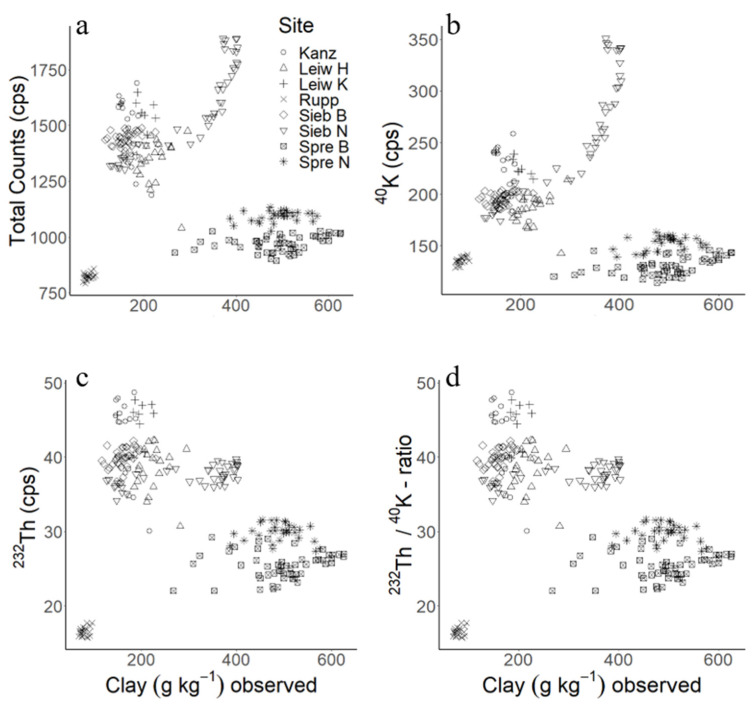
Clay content (g kg^−1^) versus gamma ROIs for the entire dataset: (**a**) total counts (cps); (**b**) K-40 (cps); (**c**) Th-232 (cps); (**d**) Th-232/K-40-ratios.

Figure 2 shows the clay content and the gamma ROIs K-40, Th-232, the Th-232/K-40-ratio and the gamma TC. No consistent relationship exists between the gamma ROIs and the clay contents at the study sites. Instead, the dataset revealed three clusters for the ROIs and clay content, which are apparently related to the geopedological situation of the individual vineyards.

While the two vineyards near Sprendlingen and the vineyard in Ruppertsberg formed individual point clouds, the remaining vineyards in Kanzem, Leiwen, and Siebeldingen (B and N) were clustered in one cloud. Here, a strong influence of the geopedological settings on gamma information and hence on soil texture becomes visible. The soils at Sprendlingen developed from Oligocene marine marl, a material that neither occurs at the other study sites nor in their river catchments. Leiwen and Kanzem are both located in the Rhenish Massif, which builds up from Lower Devonian sedimentary rock. However, the soils at Leiwen developed in Pleistocene fluvial sediments from the Moselle middle terrace that cover the hard rock; these sediments stem large amounts from the Rhenish Massif. Furthermore, the Moselle brings along sediments from the Saar, which explains the close relationship between the data from Kanzem and Leiwen (Figure 2) despite the different genesis of parent materials. Finally, the soils at Ruppertsberg were formed by Pleistocene fluvial deposits mainly originating from the Palatine Forest (Rotliegend to Bunter Sandstone sedimentary rock). These sandy sediments reveal low gamma counts with small variation. Although the individual conditions are different, the results are in line with earlier studies [12,15,34], pointing to an impact of the geopedological environment on gamma spectra.

### 3.2. Calibrating Site-Independent Prediction Models for Soil Clay Content

As a consequence of the large variation and total intensity of gamma counts from the different sites, a linear regression approach was not feasible for a site-independent calibration. Furthermore, low variation in ROIs within some vineyards (e.g., Ruppertsberg, Leiwen K, Kanzem) additionally prohibited a classical approach for site-specific calibrations via linear regression. On the other hand, the level as well as the contrasting variation in the individual ROIs between the different vineyards under study may enhance the performance of non-linear ML calibration methods because all ROIs can be included in model calibration and hence serve as predictor variables.

In Table 3, the site-independent calibration results of the repeated 10-fold CV as well as the TSV of clay content are shown. All ML methods revealed satisfactory model robustness for the calibration dataset with RPIQcv ranges from 3.78 (KNN) to 8.65 (RF). The RF approach additionally revealed the lowest RMSE_CV_ (36.8 g kg^−1^) and the best R^2^_CV_ (0.96) [35].

**Figure 3:**
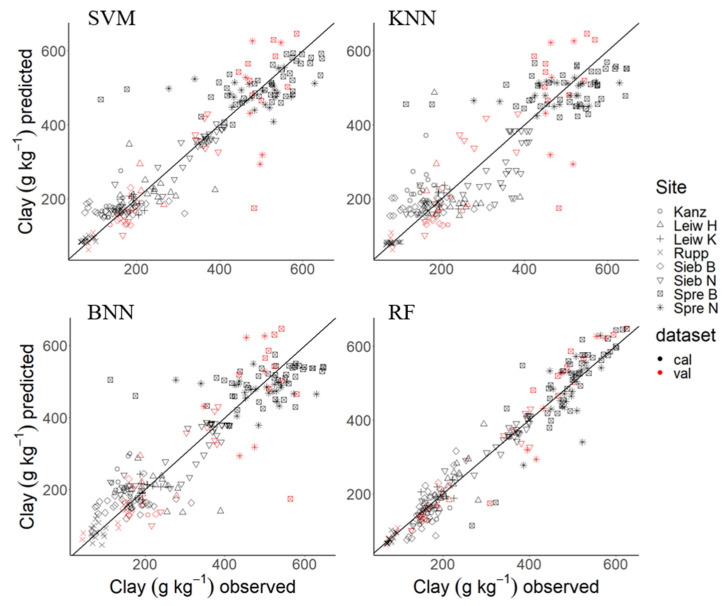
Predicted and observed values of cross-validation (cal) and test set validation (val) for clay content from site-independent regression models. SVM: Support Vector Machine; KNN: k-Nearest Neighbor, BNN: Bayesian regularized neuronal network; RF: Random Forest.

For TSV (prediction), the excellent RF model performance was confirmed with RPIQ_Pr_ (4.64), prediction error (RMSE_Pr_ = 56.7 g kg^−1^), and R^2^_Pr_ = 0.87, thus outperforming clay prediction with the other ML algorithms (Table 3 [35]). These results are also reflected in the predicted vs. observed plots for the individual ML approaches (Figure 3). Here, SVM, KNN, and BNN showed larger variation around the 1:1-line, especially in the mid-range of clay content from 300–500 g kg^−1^, as compared to RF, CV, and TSV.

Compared to more straightforward approaches, complex ML methods such as BNN or SVM have a high number of parameters to fit. In order to fit them correctly, the dataset size is an important factor [36]. However, simpler approaches such as KNN may be superior to more complex ones such as SVM, BNN, and RF when the dataset is rather small [17]. However, these statements obviously do not apply to the results of this study. Here, the dataset was rather small; nevertheless, KNN revealed the worst prediction results among the tested methods. Yet, there is no clear rule on how large the dataset size needs to be because it always depends on the complexity of the underlying problem [18]. While [11] successfully used SVM to calibrate site-independent prediction models for 10 arable fields and a rather small dataset (n = 291 for calibration), SVM calibrations were outperformed by RF in this study. These results are supported by those of other authors [25,37], who found similar results when comparing the ML methods for texture prediction, where RF calibrations showed the most reliable results. In contrast to this study, those results were not built on GS as a basis for calibration; instead, they used a number of environmental covariables including remote sensing data. Here, the RF approach yielded the best results for the limited information for GS model calibration. This underlines the suitability of RF regression for site-independent GS calibration on clay content with a limited amount of reference soil samples.

### 3.3. Model Applicability for the Site-Specific Prediction of Clay Content

The aim of a site-independent model is an appropriate texture prediction at a given site that is capable of capturing a given in-field soil heterogeneity. For this site-specific evaluation, the best model, i.e., calibrated by RF, was chosen. Therefore, the regression parameters of the site-independent RF model were evaluated for each vineyard individually. Here, the RF model revealed variable results (Table 4). The model performed well for the vineyards Sieb B, Sieb N, Spre B, and Rupp with R^2^ = 0.88–0.97, RMSE = 51.1–19.0 g kg^−1^, and RPIQ = 2.06–9.24. In contrast, the vineyards Leiw, Kanz, and Spre N were predicted less satisfactorily, with low–mediocre accuracy (R^2^ = 0.40–0.79) and model robustness (RPIQ = 0.61–1.79). A trend towards better performance of the site-independent model became visible for the sites with larger heterogeneity (i.e., coefficient of variation; Table 2) of measured clay content. This is not surprising because a larger variation in target values is considered to be beneficial for regression modelling [38].

**Figure 4:**
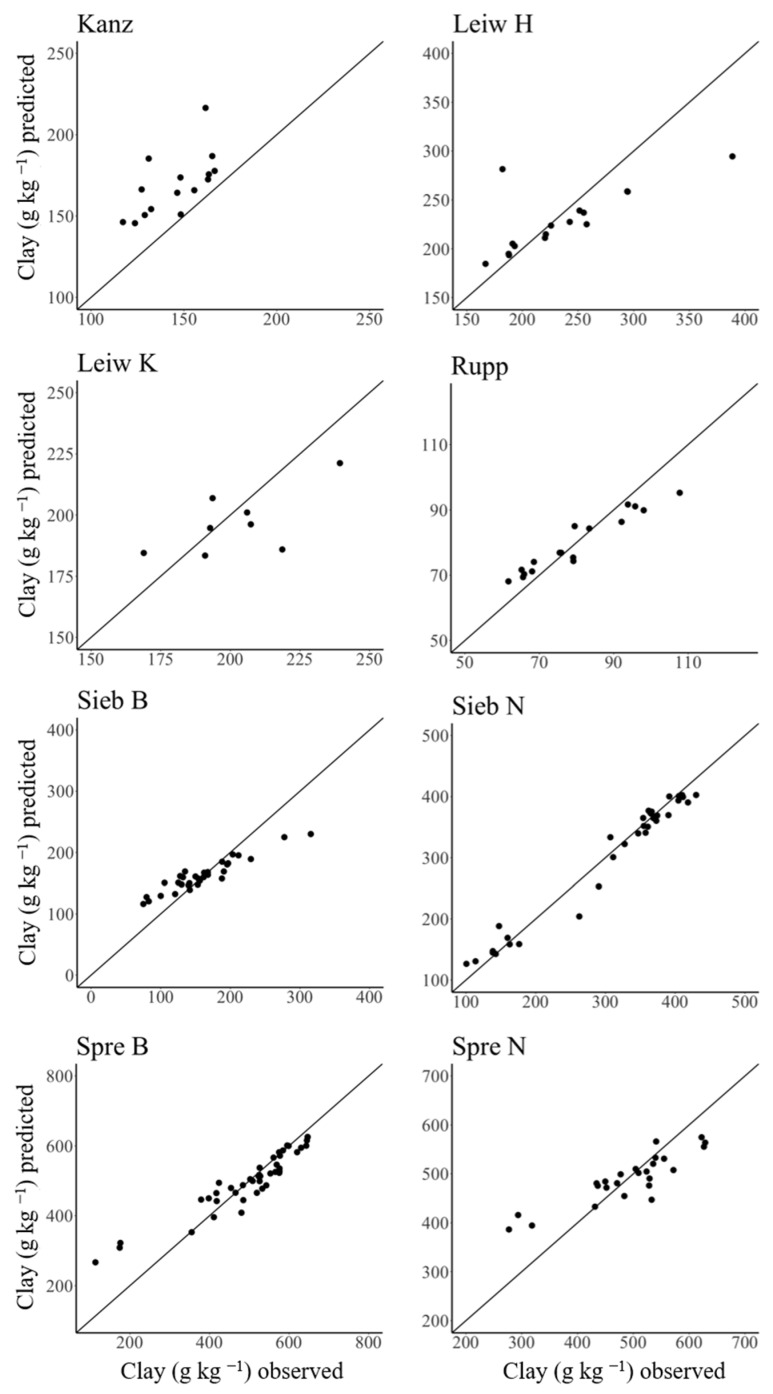
Prediction results of the site-independent RF calibration model when separately applied to individual vineyards.

Furthermore, the well-predicted vineyards contributed more samples to the entire dataset than those with poor prediction (for sample numbers, cf. Table 2). The weaker model performance for vineyards with fewer samples and smaller variations in clay content is clearly reflected in the vineyard-specific predicted vs. observed CV plots (Figure 4). All predicted values in Kanzem were too high, while the sites in Leiwen revealed large variation around the 1:1 line and hence low prediction accuracy. Figure 4 also reveals larger prediction accuracy for the vineyards in Ruppertsberg, Siebeldingen, and Sprendlingen. The intensity of the emission of the naturally occurring radionuclides in soils is highly specific for the geopedological situation, as shown by [34]. Consequently, the total amount of samples per vineyard provides weighted information for the RF algorithm on the associated geopedological setting. This most likely explains the decreased model accuracy for the small datasets from Pleistocene fluvial deposits of the Moselle region (Leiwen) and Devonian shales of the Saar region (Kanzem).

### 3.4. Applicability of Site-Independent RF Calibrations and on-the-go Measurements for Mapping the Clay Content

**Figure 5:**
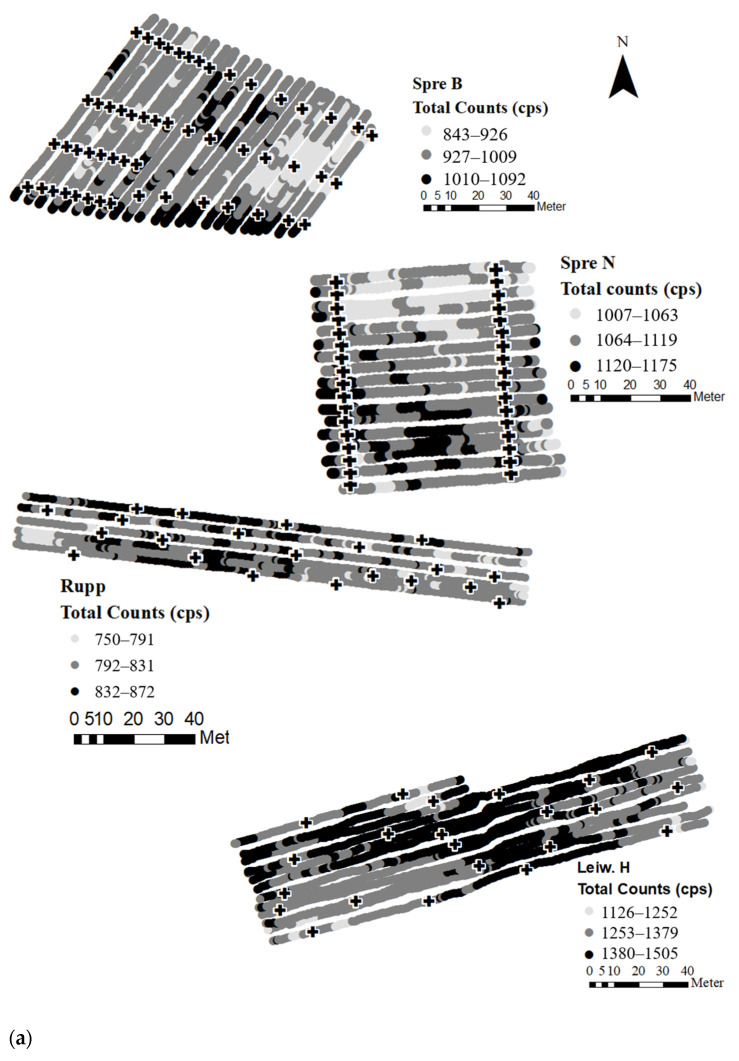

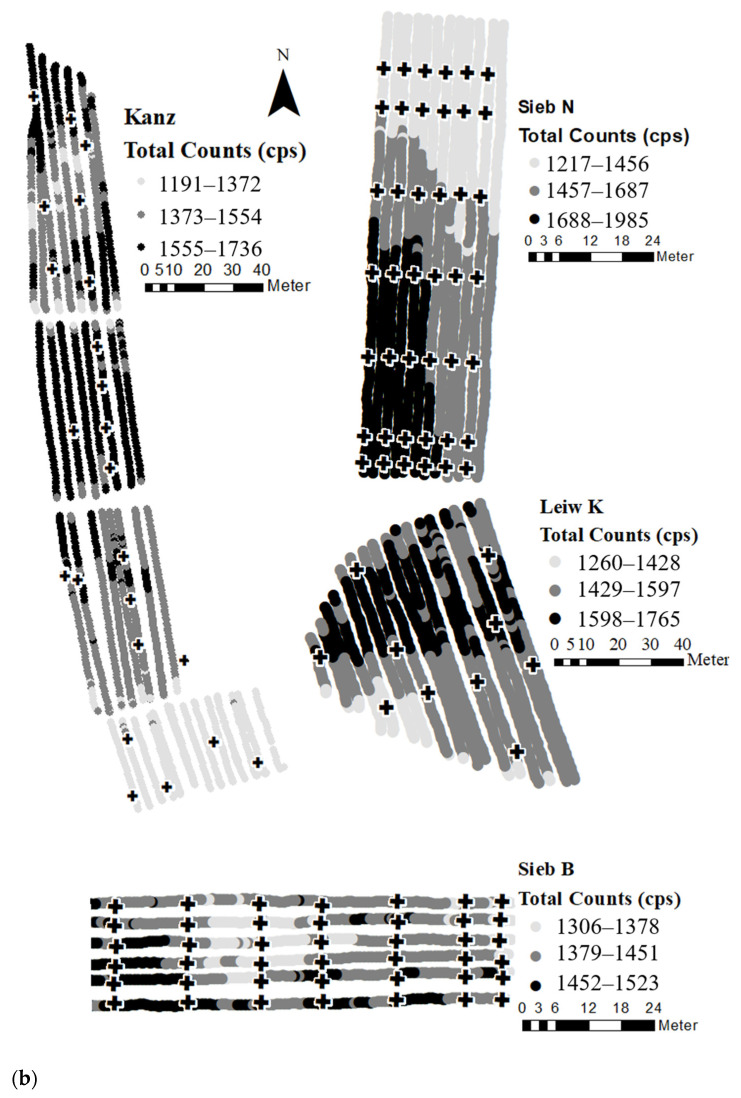
(**a**) Total counts (cps) of on-the-go gamma spectrometric measurements and ground truth sampling points of the examined vineyards. (**b**) Total counts (cps) of on-the-go gamma spectrometric measurements and ground truth sampling points of the examined vineyards.

Gamma spectrometry can be carried out in real time while driving and conducting GPS tracking. Figure 5a,b shows the moving window-smoothed TC of the examined vineyards via on-the-go measurements. Here, various sites revealed considerable in-field heterogeneity of the measured gamma signal. Therefore, the process of geostatistical data treatment, e.g., spatial interpolation, becomes potentially obsolete, if the calibrated model achieves large prediction accuracy for the parameter of interest. Low model performance would lead to poor prediction quality for each data point and, in consequence, to uncertain spatial predictions. Although site-specific prediction was not successful for all vineyards under study (see Section 3.3), the potential of an area-wide clay prediction without geostatistical data treatment, shall be evaluated.

Therefore, clay maps were created for four exemplary vineyards based on gamma spectra recorded on-the-go. These vineyards were chosen as follows: (a) good site-specific model performance and large variation in clay content (Sieb N); (b) good site-specific model performance and moderate variation in clay content (Spre B); (c) low model performance and moderate variation in clay content (Leiw H); (d) low model performance and low variation in clay content (Leiw K) (Table 4, Figure 4).

**Figure 6:**
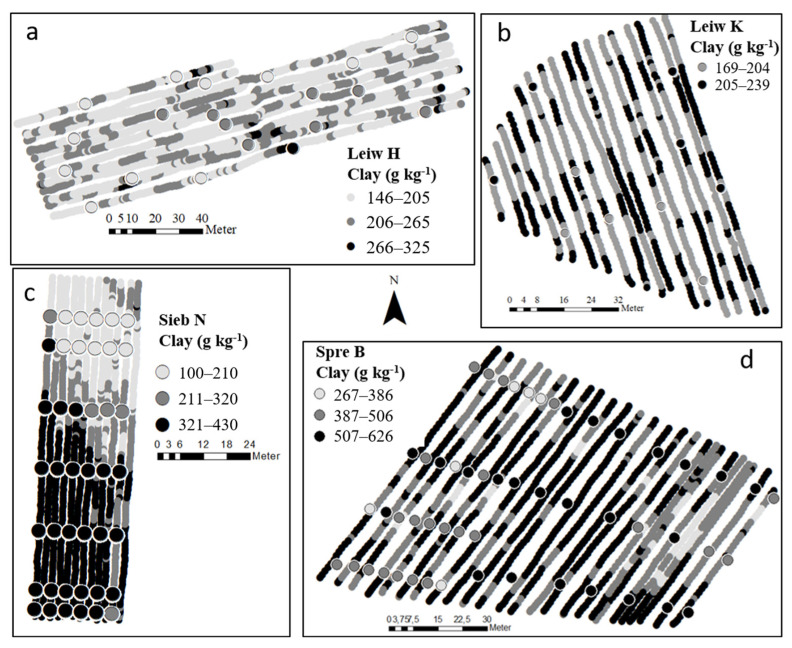
Gamma spectrometric on-the-go predicted soil clay maps of Random Forest model and ground truth data (large dots) for the vineyards in (**a**) Leiw H, (**b**) Leiw K, (**c**) Sieb N, and (**d**) Spre B.

In Figure 6, GS on-the-go maps of RF predicted soil clay content for the four selected situations are illustrated. For the vineyard Sieb N (a), the on-the-go predicted values were mostly close to the ground truth data and correctly reflect the in-field heterogeneity of soil clay content (Figure 6a). Here, a gradient in clay content from north to south is obvious. Lower clay content in the northern part of the field Sieb N is associated with an anthropogenic fill of loess as part of a slope adjustment prior to vine planting, while the southern part is dominated by clayey PPSD from Keuper weathering products. Furthermore, a transition area between the two zones in the middle of the field is characterized by loamy PPSD (weathered Bunter Sandstone). For this vineyard, RF calibration and prediction of on-the-go GS measurements correctly identified the spatial distribution of clay content in contrasting geopedological scenarios. This result clearly demonstrates the potential of on-the-go GS measurements for clay mapping by a site-independent RF calibration approach.

While the spatial distribution of clay content for the vineyard Spre B was also predicted with reliable accuracy, predictions for Leiw H and Leiw K were less successful (Figure 6).

As a result of weaker model performance, the in-field heterogeneity of clay content was not satisfactory mapped for Leiw H, and larger clay content in the middle of the field was underestimated by the model. For Leiw K, the predicted values were mostly in the range of the ground truth data. Nevertheless, low variation in classically measured clay content within this vineyard (Table 4) prevented clear spatial differentiation.

All in all, these results confirm that RF calibrations, based on GS data from different sites, can be a powerful tool for the mapping of soil clay content and its in-field heterogeneity at an individual site. Yet, some prerequisites apply: (i) sufficient model performance, (ii) enough sampling points at the site to be predicted, and (iii) a certain spatial variation in clay content. Furthermore, it should be noted that [12] found low prediction results for soils that were not included in the calibration set but originated from similar geopedological settings. Therefore, a really universal model validity remains still questionable and needs to be analyzed more detailed in future studies. It seems that enlarging the underlying database is necessary.

## 4. Conclusions

Particularly high-value crops such as vines merit a diversified management that considers small-scale soil variability, even on small plots. In the context of precision agriculture, the pronounced geopedological variability of vineyards and resulting variation in soil properties within and between the plots are a challenge. Among the various soil properties, clay content is of particular interest for vine growing. Small-scale variability of clay content should also be considered for monitoring studies, e.g., for future soil health evaluation. Consequently, there is a need for a rapid and accurate approach for the site-specific and spatially resolved evaluation of clay content. This study showed that GS in combination with RF calibration is suited for predicting clay content in a heterogeneous set of vineyards with widely varying geopedological settings. Although the calibrated model performed well for the entire dataset, the individual vineyards were predicted with variable success. For those vineyards, where the calibrated model performed well—i.e., in particular those with a pronounced heterogeneity—on-the-go GS measurements were useful in predicting the spatial distribution of clay content. This was even the case for heterogeneous geopedological settings within individual vineyards. The direct visualization of the linearly collected data for the areal characterization of the clay content (i.e., without geostatistical interpolation) does not limit the significance of the results with respect to the given data density. The origin of gamma quanta from the uppermost 30 to 40 cm implies that texture prediction from gamma spectra refers to this soil depth. However, with respect to regular deep tillage and ploughing prior to (re-) planting vines, it is assumed that texture variability of vineyard soils over depth is smaller than in arable soils. Future research should focus on enlarging the database by providing larger quantities of calibration samples—in a balanced number—from varying but thoroughly selected geopedological settings to assure a robust and universally valid calibration. This may lead to more robust performance of calibrated site-independent GS models and would optimize the use in the context of a spatially resolved clay content evaluation.

## Figures and Tables

**Table 1:** Geopedological setting of the examined vineyards.

Site	Wine Growing Region	Geopedological Setting
Kanz	Saar	Devonian sand, silt, and clay stones
Leiw H	Moselle	Pleistocene fluvial deposits from Moselle middle terrace
Leiw K	Moselle	Fluvial deposits over gravel from the Moselle middle terrace
Rupp	Palatinate	Pleistocene fluvial deposits from sandy Rotliegend to Bunter Sandstone sedimentary rock
Sieb B	Palatinate	Pleistocene loess
Sieb N	Palatinate	Pleistocene periglacial slope deposits (ppsd) mainly from loamy to clayey substrates from Keuper/Bunter Sandstone sedimentary rock. Artificial Loess deposits in northern part of the field
Spre B	Rhine-Hesse	Oligocene marl
Spre N	Rhine-Hesse	Oligocene marl

**Table 2:** Descriptive statistics of clay content as determined by conventional laboratory analysis as well as stop-and-go gamma TC’s and ROIs (cps) of the examined vineyards.

Site, Sample Number, Field Size	Stat.	Clay [g kg^−1^]	TC [cps]	K-40 [cps]	Th-232 [cps]	Th/K—Ratio
Kanz	Min.	117	1187	173.8	30	20.8
n = 18	Max.	181	1690	258.4	48.7	31.4
ca. 1 ha	Mean	148	1482	222.6	42.4	27
	coeff. var. ^1^	12.1	9.81	10.7	12.7	3.87
Leiw-H	Min.	160	1041	142.7	30.7	20.5
n = 20	Max.	389	1475	214.2	42.2	29.3
ca. 1 ha	Mean	231	1339	184.8	38.3	27.3
	coeff. var. ^1^	24	7.53	8.44	7.74	3.51
Leiw-K	Min.	169	1369	195.4	39.9	25.7
n = 10	Max.	239	1648	240.7	47.7	33.2
ca. 0.5 ha	Mean	201	1545	221.1	45	29.9
	coeff. var. ^1^	9.52	5.16	6.84	5.94	3.85
Rupp	Min.	62.1	792	129.2	15.8	13.8
n = 21	Max.	108	857	141.2	17.7	16
ca. 0.4 ha	Mean	80.2	822	135.6	16.5	15
	coeff. var. ^1^	16.9	1.87	2.51	3.61	4.07
Sieb B	Min.	75	1358	182.5	36.4	31.1
n = 40	Max.	316	1488	203.9	42.3	34.3
ca. 0.3 ha	Mean	161	1433	195	39.8	33
	coeff. var. ^1^	31.6	2.58	3.02	3.67	3
Sieb N	Min.	101	1302	174.5	34.2	0.11
n = 42	Max.	430	1890	351	39.8	0.21
ca. 0.4 ha	Mean	309	1590	259.5	27.9	0.15
	coeff. var. ^1^	32.4	12.7	22.5	3.58	21.1
Spre B	Min.	113	895	114.5	22	20.1
n = 53	Max.	647	1027	145.7	29.2	26.9
ca. 1 ha	Mean	505	970	130.1	25.4	23.8
	coeff. var. ^1^	22.5	3.59	6.68	7.19	6.46
Spre N	Min.	277	1026	139.4	27.4	21.9
n = 31	Max.	629	1134	163.5	31.6	28.4
ca. 0.6 ha	Mean	483	1093	152.7	29.8	25
	coeff. var. ^1^	18.5	2.21	4.37	4.05	5.07

^1^ coeff. var.: coefficient of variation.

**Table 3:** Site-independent 10-fold cross-validation (CV) and test set validation (TSV) results for prediction of clay content in vineyard soils based on stop-and-go gamma measurements and Support Vector Machine (SVM), k-Nearest Neighbor (KNN), Bayesian regularized neuronal network (BNN), and Random Forest (RF) regression models [35].

Model	Cross-Validation			Test Set Validation		
	RMSE_CV_ [g kg^−1^]	R^2^_CV_	RPIQ_CV_	RMSE_Pr_ [g kg^−1^]	R^2^_Pr_	RPIQ_Pr_
SVM	62.3	0.87	4.77	80.8	0.80	4.02
KNN	78.5	0.79	3.78	91.7	0.75	3.54
BNN	72.2	0.83	4.11	93.2	0.74	3.48
RF	36.8	0.96	8.65	57.6	0.87	4.64

**Table 4:** Prediction results of RF cross-validation for clay content of each vineyard.

Site	RMSE [g kg^−1^]	R^2^	RPIQ
Kanz	27.9	0.4	0.61
Leiw H	38.6	0.55	1.69
Leiw K	15.9	0.42	1.12
Rupp	5.5	0.92	4.39
Sieb B	28.9	0.89	2.06
Sieb N	19.0	0.97	9.24
Spre B	51.1	0.88	2.27
Spre N	52.4	0.79	1.72

## Data Availability

The data presented in this study are only accessible on request from the corresponding author in order to ensure the promised data protection for the private vine growers.
